# Long-term effect of municipal solid waste compost on the recovery of a potentially toxic element (PTE)-contaminated soil: PTE mobility, distribution and bioaccessibility

**DOI:** 10.1007/s11356-023-30831-y

**Published:** 2023-11-18

**Authors:** Antonio Giandonato Caporale, Carlo Porfido, Pier Paolo Roggero, Anna Di Palma, Paola Adamo, Maria Vittoria Pinna, Giovanni Garau, Matteo Spagnuolo, Paola Castaldi, Stefania Diquattro

**Affiliations:** 1 Department of Agricultural Sciences, University of Naples Federico II, Via Università 100, Portici, 80055 Naples, Italy; 2 Department of Soil, Plant and Food Sciences, University of Bari “Aldo Moro”, Via G. Amendola 165/A, 70126 Bari, Italy; 3 Dipartimento Di Agraria, University of Sassari, Viale Italia 39, 07100 Sassari, Italy; 4 Nucleo Di Ricerca Sulla Desertificazione, University of Sassari, Viale Italia 39, 07100 Sassari, Italy; 5 Research Institute On Terrestrial Ecosystems, National Research Council (IRET-CNR) Monterotondo Scalo, Rome, Italy

**Keywords:** Ageing, Organic amendments, Sequential extraction, Micro-XRF maps, In vitro bioaccessibility, Risk assessment

## Abstract

Compost from municipal solid waste (MSWC) can represent a resource for the environmental management of soils contaminated with potentially toxic elements (PTEs), since it can reduce their mobility and improve soil fertility. However, the long-term impact of compost on soil recovery has been poorly investigated. To this end, the influence of a MSWC added at different rates (i.e. 1.5, 3.0 and 4.5% w/w) to a multi-PTE-contaminated (e.g. Sb 412 mg kg^−1^, Pb 2664 mg kg^−1^ and Zn 7510 mg kg^−1^) sub-acidic soil (pH 6.4) was evaluated after 6 years since its addition. The MSWC significantly enhanced soil fertility parameters (i.e. total organic carbon, Olsen P and total N) and reduced the PTE labile fractions. The distribution maps of PTEs detected through µXRF analysis revealed the presence of Zn and Pb carbonates in the amended soils, or the formation of complexes between these PTEs and the functional groups of MSWC. A higher oral, inhalation and dermal bioaccessibility of each PTE was detected in the soil fine-grained fractions (< 2 and 2–10 µm) than in coarse particles (10–20 and 20–50 µm). The MSWC amendment generally did not modify the PTE bioaccessibility, while the relative bioaccessibility of cationic PTEs was greater than that of anionic ones (e.g. Cd > Zn > Pb > Sb > As). Pb and Sb showed the highest hazard quotients (e.g. 2.2 and 10 for Sb and Pb, respectively, in children). Overall, the results indicated that the MSWC used can be an effective option for the recovery of PTE-contaminated soils, even in the long term.

**Supplementary Information:**

The online version contains supplementary material available at 10.1007/s11356-023-30831-y.

## Introduction

Due to rapid industrial and economic development worldwide, soil contamination by potentially toxic elements (PTEs) is an increasingly severe environmental problem (Kumpiene et al. 2017). Potentially toxic elements such as lead (Pb), cadmium (Cd), zinc (Zn), arsenic (As) and antimony (Sb) are released into the soil by various anthropogenic activities including mining, waste disposal, improper fertiliser use, chemical production and industrial emissions (Yang et al. 2018). In particular, the soil contamination due to the lack of securing of abandoned mining sites, which is responsible for the spread of tailings and mining waste containing high concentrations of PTEs, is common to many countries (Khelifi et al. 2021). Specifically, Sardinia (Italy) was one of the most important mining regions in Italy (Cidu et al. 2012). In the Pb–Zn-Ag-Sb mine of Argentiera (Sassari), minerals such as sphalerite [(Zn,Fe)S], stibnite (Sb_2_S_3_), galena (PbS) and silver-rich galena [(Pb,Ag)S] were extracted for about two centuries. The land disposal of tailings and mining waste led to a historical accumulation of PTEs in this area (ISPRA 2022). The concentrations of PTEs detected in the area by previous studies, e.g. Sb ~ 109 mg kg^−1^, Pb ~ 1 215 mg kg^−1^, Zn ~ 5400 mg kg^−1^ and Cd ~ 23 mg kg^−1^ (Garau et al. 2017), exceeded the limits set by the Italian legislation for both green and agricultural areas, as well as for industrial ones (Gazzetta Ufficiale 2006).

When excess amounts of PTEs reach the soil, adverse effects on its quality were observed (Yang et al. 2018), since these inorganic contaminants are highly toxic, and show long-term persistence in soil (Ali et al. 2019). The exposure to PTEs can pose risks to human health (Pierart et al. 2015) and plant growth, as well as to the abundance, diversity and activity of soil microorganisms (Garau et al. 2017). Inhalation and ingestion of dust, e.g. emitted from dismissed mining sites, are the two main exposure pathways to PTEs for the population living near mining areas (Khelifi et al. 2021). PM_2.5_ and PM_10_ emitted by mining waste and tailings have been classified as Group 1 carcinogens by the International Agency for Research on Cancer (IARC 2016).

The European environmental regulatory framework (Directive 2004/35/EC) defines risk according to the total concentration of a chemical in soil. However, it would be more appropriate for risk assessment to consider the mobile or potentially bioavailable fraction of each contaminant (i.e. the fraction that can be transferred from soil to a living organism) and/or its bioaccessible fraction (i.e. the fraction that can be released from soil into the respiratory and gastrointestinal tracts or being absorbed by the skin of humans and animals, becoming bioavailable only if transferred into the blood; Kumpiene et al. 2017). This would avoid over- or underestimation of risk (Cipullo et al. 2018; Li et al. 2014b). Assessing the mobility and bioaccessibility of PTEs requires specific analytical protocols. Sequential extractions are methods that can be applied to different soil types to investigate PTE mobility, i.e. to identify and quantify different species, forms or phases of a contaminant in soil (Cipullo et al. 2018; Wenzel et al. 2001). Different PTE bioaccessibility protocols have been used in order to assess in vitro: tracheobronchial upper respiratory tract bioaccessibility (artificial interstitial fluid or Gamble’s solution; Moss 1979) and Gamble’s solution (GAM, pH 7.4), pulmonary bioaccessibility (artificial lysosomal fluid procedure; Stopford et al. 2003), gastrointestinal bioaccessibility (Unified BARGE Method; Barge-Ineris 2010) and dermal bioaccessibility (simulated acid and neutral skin liquids; Chaparro Leal et al. 2018). The data obtained from the assessment of various types of bioaccessibility, together with the physicochemical properties of the soil and the distribution and mobility of contaminants, can provide accurate information, useful to predict the actual risk associated with a PTE-contaminated soil (Cipullo et al. 2018). However, to the best of our knowledge, very few studies addressed both aspects (e.g.Diquattro et al. 2021a; Khelifi et al. 2020; Manzano et al. 2020).

In the last decade, the environmental management for the in situ recovery of PTE-contaminated soils is mainly based on their immobilisation, which is achieved through the addition of amendments or sorbents able to reduce the mobility of the contaminant and thus the risk of groundwater contamination, plant uptake and exposure to living organisms (Caporale et al. 2018; Diquattro et al. 2021a). Organic amendments (e.g. compost, biochar, farmyard manure and peat moss) not only promote the reduction of PTE bioavailability and soil functionality (e.g. Diquattro et al. 2021b; Garau et al. 2019; 2021; Ghani et al 2022; Nawab et al. 2019), but they can reduce the health risk index for adults and children (Ghani et al. 2022; Nawab et al. 2018), as well as increase plant biomass production (Ghani et al 2022; Nawab et al. 2018). A widely used soil amendment is compost obtained from municipal solid waste (MSWC), which is usually rich in humified organic matter and functional groups (e.g. carboxyl, phenolic hydroxyl, carbonyl and amine groups) able to complex PTEs, decreasing both their mobility and bioaccessibility in soil (Diquattro et al. 2018; Garau et al. 2019). MSWC is also useful at improving the chemical and (micro)biological functionality of soil by providing nutrients, increasing their retention in soil and stimulating plant/microbial growth and activity (Garau et al. 2017; 2019). Although MSWC revealed effective at stabilising PTEs in mining areas of different countries (Eissa et al. 2022; El Rasafi et al. 2023; Hammond et al. 2022; Laghlimi et al. 2022; Sarathchandra et al. 2022), this has been mostly proved in short-term experiments (e.g. Dominguez et al. 2019; Garau et al. 2017; Picariello et al. 2021), while little or no work investigated its long-term effect on the mobility (Asemaninejad et al. 2021; Fang et al. 2018; Hammond et al. 2022), distribution and/or bioaccessibility of PTEs in contaminated soils.

Additionally, in order to gain a more complete picture of the action of an amendment, it is also important to evaluate its action under field conditions. However, the long-term effect of MSWC on the mobility, distribution and bioaccessibility of PTEs (Sb, As, Cd, Pb and Zn in particular), under field conditions, has not been satisfactorily evaluated to date. Our hypothesis was that the addition of compost can provide long-term mitigation effects of soil PTE contamination and that such information can be useful for risk assessment over time of PTE-contaminated soils treated with compost. The objectives of this study were (i) to assess the long-term mobility, in open field conditions, of As, Sb, Cd, Pb and Zn in a MSWC-treated and untreated mining soil by means of sequential extraction procedures; (ii) to evaluate, in the same soils, the distribution of PTEs in the different particle size fractions by means of micro-XRF maps and (iii) to estimate the potential health and environmental risks by quantifying the oral, inhalation and dermal bioaccessible fractions of PTEs in the different particle size fractions of the treated and untreated soil.

## Material and method

### Study area, experimental setup and influence of MSWC on selected soil chemical properties

The study area (approx. 2 ha) was within the abandoned mining site of Argentiera (40° 44′ 11″N, 8° 08′ 52″E) in northwest Sardinia (Italy). The site is characterised by the presence of silver-rich galena [(Pb,Ag)S], sphalerite (ZnS), boulangerite (Pb_5_Sb_4_S_11_), tetrahedrite [(Cu,Fe)_12_Sb_4_S_13_], freibergite [(Ag,Cu,Fe)_12_(Sb,As)_4_S_13_], bournonite (PbCuSbS_3_) and pyrargyrite (Ag_3_SbS_3_) (Pirri 1996).

The experimental field trial, which started in January 2015, covered an area of approximately 20,000 m^2^, which was divided into 12 sub-plots (each of approximately 1600 m^2^; Fig. S1). Four treatments were conducted: untreated control soil (Control), soil treated with 1.5% MSWC w/w (MSWC 1.5%), soil treated with 3.0% MSWC w/w (MSWC 3.0%) and with 4.5% MSWC w/w (MSWC 4.5%). Each treatment was repeated in three sub-plots. The application of MSWC took place at a depth of 0–30 cm. The percentages of MSWC added were chosen because they were conventionally used in similar trials (Liu et al. 2012; Paradelo et al. 2011) and because they were actually feasible in the open field. The compost used in the experiment was produced by the Verde Vita Srl industrial composting plant (Sassari, Italy), starting from the organic fraction of municipal solid waste (MSW) and green waste; compost characteristics are shown in Table S1 (Diquattro et al. 2018). The MSWC was produced through accelerated bio-oxidation of the wet fraction within reactors equipped with a forced aeration system and a turning/transfer system using sub-vertical axis augers, in a closed building. The production of MSWC took 90 days.

In January 2021, five soil samples (2 kg each) were collected in each plot (0–30-cm depth) with an Edelman auger following a W scheme (a total of 60 soil samples were collected). Afterwards, soil samples from each plot were pooled together to obtain 12 composite samples which were used for the soil analyses.

Each composite soil sample was initially dried at room temperature and after thorough disaggregation and homogenisation, it was sieved to < 2 mm. According to particle-size analysis, carried out using the pipette method (Day 1965), the soil in the sampled area was a sandy loam (USDA texture classification) with 66% coarse sand, 23.3% fine sand, 10.2% silt and 0.5% clay. Chemical analyses (i.e. electric conductivity (EC), pH, cation exchange capacity (CEC), extractable P and active carbonates) were determined according to the Italian Official Methods (Gazzetta Ufficiale 2002). Total organic carbon (TOC) and total N were determined using a CHN analyser (Leco CHN 628) and Soil LCRM Leco part n° 502–697 as calibration sample; dissolved organic carbon (DOC) was estimated as previously described by Brandstetter et al. (1996). The pseudo-total concentration of PTEs (PS-TOT) in soils was quantified, after digestion with HNO3 and HCl (ratio 3:1 v/v) in a Milestone UltraWAVE SRC Technology, using a Perkin Elmer AAnalyst 200 flame atomic absorption spectrometer (FAAS) for Zn and a Perkin Elmer AAnalyst 400 equipped with HGA 900 graphite furnace (GFAAS) for Pb, Cd, As and Sb. A standard reference material (NIST-SRM 2711A) was included for quality control.

### PTE distribution in the soil particle-size fractions

Soil samples unamended and amended with MSWC, dried and sieved to < 2 mm, were physically separated in particle-size fractions (< 2, 2–10, 10–20, 20–50 and 50–2000 µm), by sonication, consecutive cycles of centrifugation (to separate the fraction < 2 µm), and then sedimentation and decantation into Esenwein cylinders (to split the coarser particles), with exhaustive recovery of the fractions after three different times calculated on the basis of Stokes’ law, according to the protocol described by Khelifi et al. (2021).

To assess PS-TOT of Sb, As, Pb, Cd and Zn, in all particle-size fractions (< 2, 2–10, 10–20, 20–50 and 50–2000 µm), 0.25 g of each composite sample (in triplicate) was (i) digested with aqua regia (HCl/HNO3, 3:1; ISO 12914, 2012) in a microwave digestion system (model StartD); (ii) analysed by inductively coupled plasma–optical emission spectrometry (ICP-OES, Thermo Scientific iCAP 7400). The European Reference Material CRM 141R was used to monitor the quality of analyses, with PTE recoveries around ± 10% of the certified values.

### Influence of MSWC on PTE mobility as assessed through sequential extraction procedures (SEP)

As and Sb mobility in the different composite soil samples was evaluated through the sequential extraction procedure (SEP) proposed by Wenzel et al. (2001) with minor modifications. This procedure was used as it is specific to elements like As and Sb which behave as anions in soil. Briefly, triplicate soil samples (1 g) from each composite sample were treated with different extraction solutions. Deionised water (Fraction 1, F1), 0.05 M (NH_4_)_2_SO_4_ (Fraction 2, F2) and 0.05 M NH_4_H_2_PO_4_ (Fraction 3, F3) were used to determine, respectively, the water-soluble fraction, the non-specifically adsorbed fraction and the specifically adsorbed fraction of Sb and As. The mobility of Pb, Cd and Zn was determined by the procedure of Basta and Gradwohl (2000), a simple and relatively fast extraction method widely used in the literature (e.g. Diquattro et al. 2021a; Garau et al. 2017; Li et al. 2014a; Smičiklas et al. 2021). The following PTE fractions were determined: water-soluble and readily exchangeable Cd, Pb and Zn (Fraction 1, F1; extracted with a 0.5 M Ca(NO_3_)_2_ solution); PTEs forming weak surface complexes (Fraction 2, F2; extracted with a 1 M NaOAc solution at pH 5.0) and surface complexed and precipitated Cd, Pb and Zn (Fraction 3, F3; extracted with a 1 M Na_2_EDTA solution).

In both procedures, after each extraction step, soil suspensions were centrifuged at 3500 rpm for 10 min. Supernatants were then collected and Sb, As, Cd, Pb and Zn concentrations determined in the liquid phase as described before for the pseudo-total concentration of PTEs. The resulting soils (after all extraction steps) were digested with reverse aqua regia (HNO_3_/HCl, 3:1; ISO 11466, 1995) and microwave mineralised using US EPA method 3051A, to quantify the residual fraction of PTEs (Fraction 4, F4). A standard reference material (NIST-SRM 2711A) was included for quality control, with PTE recoveries between 93.5 and 90% of the certified values.

### XRPD and µXRF analyses

An X-ray powder diffractometer (XRPD, MiniFlex I, Rigaku Corporation, Tokyo, Japan), equipped with a Cu tube (Cu Kα, 30 kV, 15 mA), was used for mineralogical analyses. Data were acquired between 3 and 70° 2*θ* with a step width of 0.02° 2*θ* and a counting time of 3 s/step.

A micro X-ray fluorescence spectrometer (µXRF, M4 Tornado, Bruker Nano GmbH, Germany) was used to investigate the distribution and correlation of PTEs (pseudo-total) in both unamended and amended soils. For this purpose, thin Sects. (30-µm thickness) were prepared after soil air-drying, sieving 2 mm and embedding in epoxy resin (Allegretta et al. 2018). The instrument was operated under vacuum (20 mbar) using a Rh tube X-ray source (50 kV, 600 µA, 30 W, spot size of 25 µm) with polycapillary optics and two 30 mm2 XFlash® silicon drift detectors. Element distribution maps (10 mm × 30 mm) were collected with a step size of 25 µm and an acquisition time of 10 ms/pixel, in “3 frame” mode to increase the signal-to-noise (S/N) ratio. In order to get more accurate distribution maps of certain elements (e.g. Pb), acquisition was repeated using the AlTiCu filter.

### Oral, lung and dermal bioaccessibility of PTEs

The medium-fine particle-size fractions (< 2, 2–10, 10–20 and 20–50 µm) of soil samples unamended and amended with MSWC were extracted by (i) Unified BARGE Method (UBM) (Barge-Ineris 2010), to assess the gastric (G, pH 1.2) and gastrointestinal (GI, pH 6.3) PTE bioaccessibility; (ii) Artificial Lysosomal Fluids (ALF) (pH 4.5; Stopford et al. 2003) and Gamble’s solution (GAM, pH 7.4; Moss 1979), to evaluate PTE bioaccessibility in the upper and lower respiratory tracts, respectively and (iii) simulated acid (NIHS, pH 4.7) and neutral (CEN, pH 6.5) skin liquids secreted from eccrine and apocrine glands, respectively, to study the dermal bioaccessibility of PTEs (Chaparro Leal et al. 2018). All extractions were carried out in a shaking thermostatic water bath at 37 ± 1 °C under stirring (80 oscillations min^−1^), with a solid/solution ratio of 1:37.5 (G-UBM), 1:97.5 (GI-UBM), 1:50 (ALF), 1:100 (GAM) and 1:25 (NIHS and CEN), for 1 h (G-UBM), 1 + 4 h (GI-UBM), 24 h (ALF and GAM) and 8 h (NIHS and CEN) on the basis of physiological residence time or exposure duration (Khelifi et al. 2021). The coarsest particle-size fraction (50–2000 µm) was not included in the bioaccessibility tests, because it easily undergoes sedimentation and does not reside in the air long enough to pose a risk to human health (Schaider et al. 2007).

The bioaccessibility of the PTEs in the particle-size fractions was shown and discussed in terms of (i) bioaccessible amounts (mg of each PTE per kg of a soil fraction) and (ii) relative bioaccessibility (RB) (i.e. the bioaccessible amount of each PTE in a soil fraction expressed as percentage of the total content of the PTE in the same fraction).

### Risk assessment

The content of bioaccessible PTEs extracted from the medium-fine particle-size fractions (< 50 µm) of the soils amended and unamended with MSWC was used to quantify the non-carcinogenic (NCR) and carcinogenic (CR) risks for human health following a U.S.EPA–based risk assessment assay (i.e. U.S.EPA 2011). These risks are related to the bioaccessibility of PTEs resulting from oral, inhalation and dermal exposure, which can arise from the suspension of soil medium-fine particle-size fractions in the air (< 50 µm, oral and dermal exposure; < 10 µm, inhalation exposure). The equations, parameters and reference values applied in the risk assessment are summarised and shown in the Supplementary text and Table S5.

### Statistical analysis

All the analyses were carried out on triplicate soil samples collected from each composite soil, and mean values ± standard errors were reported in tables and figures (except for bioaccessibility data for which only mean values were reported).

A one-way analysis of variance (one-way ANOVA) was used to evaluate the effect of increasing concentration of MSWC on the soil chemical characteristics and PTE mobility. When significant *p*-values (*p* < 0.05) were obtained, mean multiple comparisons were performed with the post hoc Tukey test. The statistical analyses were performed using the SigmaPlot software (SPSS Inc., Chicago, IL, USA).

The data on the distribution and bioaccessibility of PTEs in the soil particle-size fractions and values from US EPA risk assessment were analysed according to a factorial combination of four/five particle-size fractions (PS) and four different compost rates (CR). The analysis of variance was therefore conducted as two-way ANOVA using the software package IBM SPSS Statistics v26 (SPSS Inc., Chicago, IL, USA). When mean separation was required, it was conducted through Duncan’s multiple range test (DMRT), performed at *p* < 0.05.

Prior to the ANOVA tests, the Cochran and Shapiro–Wilk tests were performed to test homoscedasticity and normality, respectively, and in case of violation, the ANOVA was performed on log-transformed data.

## Results and discussion

### Influence of MSWC on selected soil chemical properties

The control soil was weakly acidic (pH 6.36) and had an organic matter content of approximately 2%, a very low concentration of total N and extractable P and a low CEC, compared to the average fertility values of Italian agricultural soils (Costantini 2006; Table 1). After 6 years since MSWC addition, the effect of the amendment on the soil chemical properties was still significant. Indeed, the addition of MSWC caused a significant increase of the soil pH which was depending on its rate (i.e. + 0.3, + 0.8 and + 0.9 pH units in soils amended with 1.5, 3.0 and 4.5% MSWC, respectively). This was probably due to the alkaline nature of compost (Table S1; Erana et al. 2019). Similarly, Asemaninejad et al. (2021) observed a slight increase in pH (~ + 0.4) in a neutral soil (pH 7.1) amended with compost after 4 and 10 years since its addition. As expected, significant increases in TOC (e.g. + 1.6% times in 4.5% MSWC) and DOC (e.g. + 5.0 times in 4.5% MSWC) were observed in MSWC-amended soils (Table 1). This result is particularly significant, since previous studies reported a reduction of TOC and DOC after several years since the addition of compost (Asemaninejad et al. 2021; Picariello et al. 2021). Also, the CEC increased in MSWC-amended soils, as well as the extractable P and total N (Table 1). The pseudo-total concentrations of As, Cd, Pb, Sb and Zn did not vary in the different soil samples, but exceeded by 1.3, 10.2, 26.6, 41.2 and 300 times, respectively, the threshold concentrations for agricultural soils imposed by Italian regulations (Decree, Ministerial (DM) 2019; Table 1).

**Table 1:** Selected physicochemical characteristics of control and MSWC-amended soils (mean ± standard error)

Soil parameter	Control	MSWC 1.5%	MSWC 3.0%	MSWC 4.5%
pH_H2O_	6.36 ± 0.07^c^	6.63 ± 0.05^b^	7.13 ± 0.07^a^	7.23 ± 0.07^a^
EC (dS m^−1^)	2.20 ± 0.06^a^	0.81 ± 0.04^b^	0.44 ± 0.08^c^	0.33 ± 0.01^c^
Organic matter (%)	2.11 ± 0.07^c^	2.64 ± 0.14^b^	2.78 ± 0.06^b^	3.66 ± 0.22^a^
Total organic C (%)	1.35 ± 0.04^c^	1.59 ± 0.08^b^	1.67 ± 0.04^b^	2.26 ± 0.09^a^
DOC (mg g^−1^)	0.01 ± 0.00^c^	0.03 ± 0.00^b^	0.03 ± 0.00^b^	0.05 ± 0.00^a^
Total N (‰)	1.10 ± 0.00^b^	1.94 ± 0.01^a^	1.97 ± 0.01^a^	1.69 ± 0.01^a^
Active carbonate (g kg^−1^)	4.32 ± 0.40^a^	4.14 ± 0.21^a^	4.11 ± 0.41^a^	3.29 ± 0.55^a^
Extractable P (mg kg^−1^)	0.33 ± 0.02^c^	3.35 ± 0.27^b^	4.34 ± 0.08^a^	3.48 ± 0.36^ab^
CEC (cmol_( +)_ kg^−1^)	4.64 ± 0.37^b^	12.8 ± 0.71^a^	12.9 ± 0.32^a^	13.9 ± 0.63^a^
*Total PTEs (mg kg*^−1^*)*				
As	38.2 ± 1.19^a^	34.8 ± 1.18^a^	35.5 ± 0.90^a^	37.3 ± 1.26^a^
Cd	50.8 ± 2.84^a^	50.5 ± 2.89^a^	48.5 ± 2.67^a^	40.7 ± 2.99^a^
Pb	2664 ± 39.1^a^	2658 ± 30.7^a^	2660 ± 75.4^a^	2599 ± 66.4^a^
Sb	412 ± 25.5^a^	411 ± 17.9^a^	409 ± 16.2^a^	411 ± 16.2^a^
Zn	7510 ± 274^a^	7554 ± 322^a^	7569 ± 287^a^	7545 ± 287^a^
USDA texture	Coarse sand soil			

Different letters in a line indicate statistically significant differences according to Tukey test (*p* < 0.05)

Although previous studies (e.g. Fagnano et al. 2011; Huang et al. 2016) recommended an annual addition of compost to the soil, our results show the effectiveness of a single application over a medium to long term. The addition of MSWC increased soil fertility (e.g. organic matter and CEC) and the concentrations of essential plant nutrients (N total and extractable P), creating the preconditions for the establishment of a permanent plant cover. This represents a necessary key step in restoring the ecological functionality of PTE-contaminated soils characterised by a poor fertility status.

### PTE distribution in the soil particle-size fractions

The physical separation of control and MSWC-amended soil samples in particle-size fractions (< 2, 2–10, 10–20, 20–50 and 50–2000 µm) evidenced a large occurrence of coarse sand particles (50–2000 µm), representing 77–81% of the total weight (Fig. S2), and thus, revealing the coarse-textured nature of this soil. The remaining fractions were quite uniformly distributed in fine sand (20–50 µm), course silt (10–20 µm), fine silt (2–10 µm) and clay (< 2 µm) particles. The last two fractions (< 10 µm), whose sum ranged from 10 to 14% of the total weight (Fig. S2), are the most concerning for the environment and human health, since they can contribute to airborne PM_10_ and PM_2.5_, when eroded by the wind and dispersed into the atmosphere (Khelifi et al. 2021).

The evaluation of the pseudo-total PTE concentrations in the different particle-size fractions of the mining soil samples (Tables 2 and 3) revealed, as is well known, a significant tendency of PTEs to accumulate in the finer particle-size fractions (i.e. < 2 and 2–10 µm). Soil amendment with MSWC did not produce significant differences in the PTE pseudo-total concentrations, in comparison to the control, except for Sb. This was conceivable, because soil amendment with organic matter can strongly affect the PTE bioavailability, but not their total content in soil (apart from a negligible dilution effect).

**Table 2:** Pseudo-total (PS-TOT) and bioaccessible fractions (from G to CEN) of Zn, Pb and Cd in particle-size fractions of control and MSWC amended soils, extracted by gastric (G) and gastrointestinal (GI) solutions, lysosomal (ALF) and lung interstitial (GAM) fluids, acid (NIHS) and neutral (CEN) synthetic sweats

Source of variance	PS-TOT	G	GI	ALF	GAM	NIHS	CEN
	mg kg^−1^						
Zn							
< 2 µm	18,575^a^	9627^a^	4766^a^	14,399^a^	30^a^	12,671^a^	84
2–10 µm	12,398^b^	6979^b^	3359^b^	9898^b^	13^b^	9879^a^	197
10–20 µm	9379^c^	4378^c^	2047^b^	6256^c^	10^b^	5630^b^	41
20–50 µm	11,327^bc^	4524^c^	2011^b^	6404^c^	11^b^	5211^b^	23
*Particle size (PS)*	**	***	*	***	**	**	*ns*
Control	12,900	6662	2908	9548^a^	10^c^	8946	68
MSWC 1.5%	14,123	6647	3479	9733^a^	17^b^	9168	157
MSWC 3.0%	11,157	5397	2782	7480^b^	29^a^	7010	108
MSWC 4.5%	13,520	6515	3152	9888^a^	14^bc^	7668	31
*Compost rate (CR)*	*ns*	*ns*	*ns*	*	**	*ns*	*ns*
*PS x CR*	*ns*	*ns*	*ns*	*ns*	*	*ns*	*ns*
Pb							
< 2 µm	5704^a^	2655^a^	561	3445^a^	7.3^a^	716	2.4
2–10 µm	3980^b^	2194^b^	540	2569^b^	2.8^b^	739	2.2
10–20 µm	3038^c^	1678^c^	538	1964^c^	2.7^b^	617	1.4
20–50 µm	3600^bc^	1899^bc^	641	2200^bc^	2.7^b^	560	0.8
*Particle size (PS)*	**	*	*ns*	**	***	*ns*	*ns*
Control	3778	1927	383	2331	2.1^c^	611	1.3
MSWC 1.5%	4584	2379	760	2834	3.7^b^	773	3.3
MSWC 3.0%	4135	2282	751	2585	8.0^a^	733	1.5
MSWC 4.5%	4126	2017	570	2642	3.1^bc^	562	0.9
*Compost rate (CR)*	*ns*	*ns*	*ns*	*ns*	***	*ns*	*ns*
*PS x CR*	*ns*	*ns*	*ns*	*ns*	**	*ns*	*ns*
Cd							
< 2 µm	37^a^	24^a^	17^a^	30^a^	0.24	29^a^	1.3
2–10 µm	25^bc^	17^b^	12^b^	20^b^	0.17	21^b^	2.1
10–20 µm	23^c^	13^c^	9.1^b^	16^c^	0.13	15^c^	0.7
20–50 µm	30^b^	14^c^	9.5^b^	17^bc^	0.16	16^c^	0.5
*Particle size (PS)*	*	**	*	**	*ns*	**	*ns*
Control	28	18	13	21	0.14	22	1.1
MSWC 1.5%	31	17	12	21	0.22	21	1.7
MSWC 3.0%	25	15	11	17	0.22	19	1.2
MSWC 4.5%	29	17	12	22	0.16	19	0.6
*Compost rate (CR)*	*ns*	*ns*	*ns*	*ns*	*ns*	*ns*	*ns*
*PS x CR*	*ns*	*ns*	*ns*	*ns*	*ns*	*	*ns*

For the sake of clarity, this wide table shows only the mean values, not followed by standard errors. Particle size (PS), compost rate (CR) and their interactions were compared by two-way ANOVA, Duncan’s multiple range test (*p < 0.05; **p < 0.01; ***p < 0.001; *ns* not significant). Different lowercase letters within each column indicate significant differences (*p* < 0.05)

**Table 3:** Pseudo-total (PS-TOT) and bioaccessible fractions (from G to CEN) of Sb and As in particle-size fractions of control and MSWC amended soils extracted by gastric (G) and gastrointestinal (GI) solutions, lysosomal (ALF), and lung interstitial (GAM) fluids, acid (NIHS) and neutral (CEN) synthetic sweats

Source of variance	PS-TOT	G	GI	ALF	GAM	NIHS	CEN
	mg kg^−1^						
Sb							
< 2 µm	1399^a^	25^a^	56^a^	524^a^	5.5^a^	96^a^	1.2
2–10 µm	856^b^	18^b^	52^ab^	307^b^	2.6^b^	100^a^	0.7
10–20 µm	541^c^	13^b^	44^b^	179^c^	1.9^b^	79^ab^	0.6
20–50 µm	554^c^	12^b^	45^b^	153^c^	1.8^b^	63^b^	1.0
*Particle size (PS)*	***	*	*	***	**	*	*ns*
Control	795^c^	15	41^c^	274	3.0	74^b^	0.9
MSWC 1.5%	902^a^	21	62^a^	328	2.4	108^a^	0.7
MSWC 3.0%	841^b^	20	56^ab^	284	3.1	93^ab^	0.8
MSWC 4.5%	856^b^	15	47^bc^	295	3.3	73^b^	1.0
*Compost rate (CR)*	**	*ns*	*	*ns*	*ns*	*	*ns*
*PS x CR*	**	*ns*	*ns*	*ns*	*ns*	*ns*	*ns*
As							
< 2 µm	131^a^	7.8^a^	4.0^c^	20^a^	3.4^a^	0.41	0.16
2–10 µm	83^b^	7.2^a^	7.5^a^	14^b^	1.9^b^	0.21	0.16
10–20 µm	52^c^	4.9^b^	6.6^ab^	8.6^c^	1.0^b^	0.30	0.16
20–50 µm	54^c^	4.0^b^	5.3^bc^	6.4^c^	0.9^b^	0.20	0.17
*Particle size (PS)*	**	*	*	**	*	*ns*	*ns*
Control	91	6.6	6.0	14	2.4	0.31	0.13
MSWC 1.5%	70	5.9	6.4	12	1.2	0.36	0.15
MSWC 3.0%	73	5.7	6.2	11	1.4	0.36	0.19
MSWC 4.5%	75	5.1	4.7	10	1.6	0.06	0.22
*Compost rate (CR)*	*ns*	*ns*	*ns*	*ns*	*ns*	*ns*	*ns*
*PS x CR*	*ns*	*ns*	*ns*	*ns*	*ns*	*ns*	*ns*

For the sake of clarity, this wide table shows only the mean values, not followed by standard errors. Particle size (PS), compost rate (CR) and their interactions were compared by two-way ANOVA, Duncan’s multiple range test (*p < 0.05; **p < 0.01; ***p < 0.001; *ns* not significant). Different lowercase letters within each column indicate significant differences (*p* < 0.05)

**Fig. 1:**
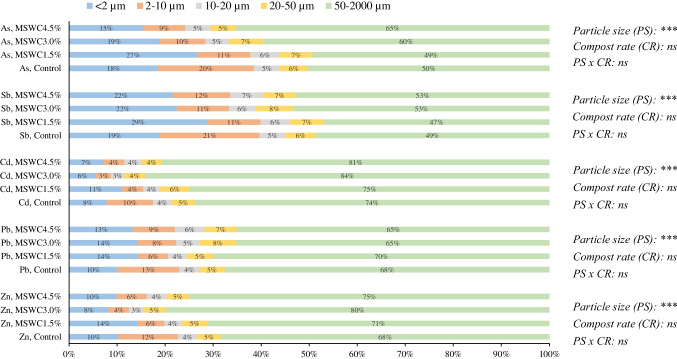
Distribution (% of the total content) of Zn, Pb, Cd, Sb and As in particle-size fractions (< 2, 2–10, 10–20, 20–50 and 50–2000 µm) of soil samples, as affected by the amendment with MSWC. Particle size (PS), compost rate (CR) and their interactions were compared by two-way ANOVA, Duncan’s multiple range test (*p < 0.05; **p < 0.01; ***p < 0.001). ns, not significant

The distribution of PTEs in the different particle-size fractions—namely the product of the element concentration in the fraction (Tables 2 and 3 and S2) for the abundance of the fraction (Fig. S2)—is shown in Fig. 1. A large proportion of PTE content (≥ 50%, Fig. 1) was distributed in the coarsest particle-size fraction (50–2000 µm; the most abundant in all the soil samples: 77–81% of the total weight, Fig. S2), containing particles which are prone to sedimentation and do not reside in the air for enough time to pose risks for human health (Schaider et al. 2007). We also observed a higher occurrence of cationic PTEs (Zn, Pb and Cd) in the coarsest fraction than that of anionic PTEs (Sb and As). However, a significant content of PTEs also accumulated in the fine particle-size fractions (2–10 and < 2 µm): on average, 18% of total Zn, 22% of total Pb, 13% of total Cd, 37% of total Sb and 32% of total As (Fig. 1). These fine particles are the greatest concern for human health, since soil particulate < 10 µm has the potential to enter in the human tracheobronchial (PM_10_) and alveolar (PM_2.5_) regions. Ajmone-Marsan et al. (2008) and Ljung et al. (2006) also observed a significant accumulation of As and Cu in fine soil particle-size fractions (< 50 µm for As; < 2 µm for Cu). A similar trend of As in gold mine tailings was described by Meunier et al. (2011). The input of MSWC into the soil at increasing rates did not significantly change the distribution of PTEs in the soil particle-size fractions (Fig. 1). However, the addition of MSWC at higher rates (3.0% and 4.5%) slightly reduced the distribution of PTEs (except Pb) in the finer soil fractions (2–10 and < 2 µm) and increased that in the coarser soil particles (50–2000 µm).

### Influence of MSWC on PTE mobility as assessed through sequential extraction procedures (SEP)

**Fig. 2:**
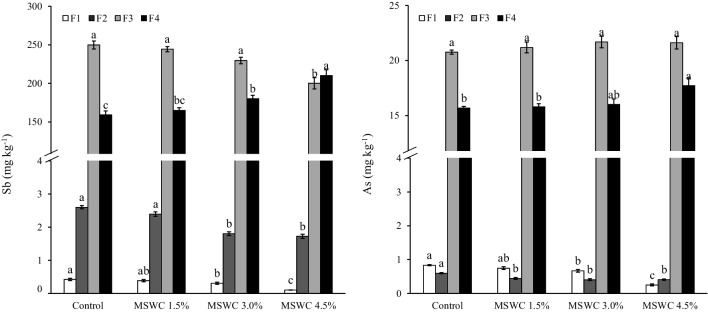
Sb and As released after sequential extraction procedure (means ± standard errors) in control and MSWC-amended soils. F denotes extraction solutions: F1 = H2O; F2 = (NH4)2SO4; F3 = (NH4)H2PO4; F4 = HNO3/HCl. Within each fraction, bars with different letters denote statistically significant differences according to the Tukey *t*-test (*p* < 0.05)

**Fig. 3:**
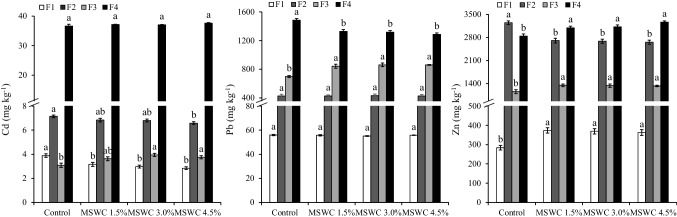
Cd, Pb and Zn released after sequential extraction procedure (means ± standard error) in control and MSWC-amended soils. F denotes extraction solutions: F1 = Ca(NO3)2; F2 = NaOAc; F3 = Na2-EDTA; F4 = HNO3/HCl. Within each fraction, bars with different letters denote statistically significant differences according to the Tukey test (*p* < 0.05)

The mobility of PTEs in the soil samples was studied through different sequential extraction procedures based on the cationic or anionic nature of the PTEs. In both procedures, F1 and F2 are the most relevant fractions from an environmental point of view, since they represent the most mobile and easily available PTE pools for plant (Figs. 2 and 3). The F1 of Sb and As in the different soils was less than 1.0 mg kg^−1^ and was significantly higher in the control soil than in the MSWC-treated ones (i.e. up to 77 and 70%, for Sb and As, respectively) (Fig. 2). The immobilising capability of MSWC can be attributed to the formation of stable interactions between the HAsO^2−^ or Sb(OH)^ˉ^ oxyanions (the dominant As and Sb species in aerobic soils) with different functional groups of compost, such as oxy-carboxylic acids and polyols (Diquattro et al. 2018, 2021b; Dominguez et al, 2019; Garau et al. 2017; Picariello et al. 2021). In addition, the formation of ternary complexes in which polyvalent metal cations (released by compost) like Ca^2+^ or Mg^2+^ acted as bridging elements between the negatively charged functional groups of MSWC and arsenate or antimonate anions, as well as the co-precipitation of the latter with metals in compost (i.e. Ca^2+^), may have contributed to the immobilisation of PTEs (Lyu et al. 2023; Pintor et al. 2021). Similarly, the Cd-F1 was significantly reduced after 6 years since compost addition (e.g. < 27% in MSWC 4.5% with respect to control), while no significant (*p* > 0.05) changes were observed for Pb-F1 (Fig. 3). On the opposite, an increase was observed for Zn-F1 in the compost amended soils (e.g. + 28% in MSWC 4.5% with respect to control; Fig. 3). This latter could be attributed to the formation of soluble complexes between Zn and DOC, as well as to exchange reactions between divalent cations present in compost (e.g. Ca and Mg) and Zn adsorbed on soil colloids (Paradelo et al. 2018).

F2 of As and Sb (i.e. the non-specifically adsorbed fraction) was lower in the amended soils compared with the control (e.g. − 33.5% for Sb and − 32.0% for As in MSWC 4.5% compared to control; Fig. 2). With regard to F3, which quantifies the Sb and As specifically adsorbed on the solid phase surface through inner-sphere complexes, a significant decrease of Sb-F3 was observed in the MSWC 4.5%-treated soil (i.e. − 20%) compared with control soil, while no changes were detected in As-F3 (Fig. 2). Similarly, Garau et al. (2019) observed a reduction of the Sb released with (NH_4_)H_2_PO_4_ in the short term (i.e. after 4 months since MSWC addition), while compost addition did not affect the release of As. The F2 of Cd and Zn decreased in MSWC-treated soils, while the F2 of Pb was not significantly influenced by the treatments (Fig. 3). The F3 increased in all amended soils: e.g. + 21, + 23 and + 15% for Cd, Pb and Zn in MSWC 4.5%, respectively, with respect to C-soil (Fig. 3). This result is a significant outcome from an environmental point of view, since this step of the sequential extraction quantifies hardly bioavailable or hardly leachable PTEs (Garau et al. 2019; 2021).

The not readily mobile PTE pool (i.e. residual fraction of Sb, As, Cd, Pb and Zn; F4) represented more than 30% of pseudo-total PTEs (Figs. 2 and 3). The residual Sb, As and Zn fractions were higher in amended soils, particularly in those which received the highest MSWC rates (e.g. > 32, 13 and 15% in MSWC 4.5%, respectively, compared to control), suggesting the occurrence of stable and long-lasting interactions between MSWC and PTEs. On the other hand, the long-term effect of the MSWC amendment did not significantly affect the residual fraction of Cd, while that of Pb was higher in the control soil (e.g. − 10.5% in the MSWC 4.5% soil). We attributed this result to the increased formation of strong complexes between Pb and organic matter in the MSWC, as evidenced by the rise of Pb extracted with Na_2_-EDTA (i.e. F3) (Ozbas and Catalbas 2021).

The experimental results suggest that the PTE-MSWC interaction mechanisms were stable in the long term and contributed to the reduction of the most environmentally hazardous fraction of PTEs. Furthermore, compost added at the highest dosage (MSWC 4.5%) was the most effective and practically applied treatment at reducing As and Sb mobility in the long term.

### Influence of MSWC on PTE distribution as assessed through XRPD and µXRF

**Fig. 4:**
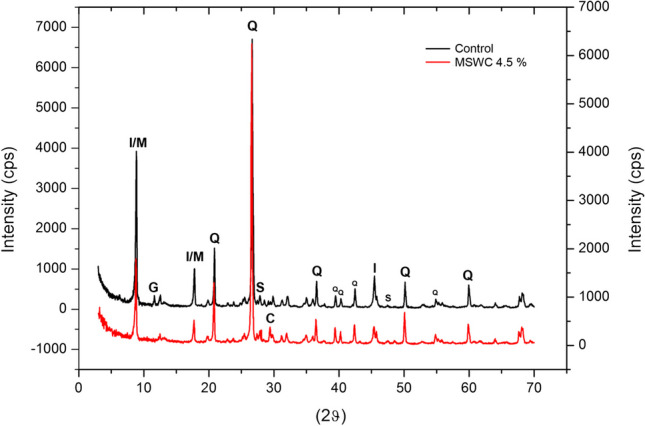
XRPD—X-ray diffraction patterns of the control (black line) and MSWC 4.5% (red line) soils. Main diffraction peaks of detected mineral phases are labelled as follows: I/M, illite/muscovite; G, gypsum; Q, quartz; S, sphalerite; C, calcite. Not labelled peaks are lower intensity diffraction peaks of the same minerals

The control and the MSWC amended soils were analysed for mineralogy. However, only at the maximum rate of amendment (e.g. MSWC 4.5%) we observed differences between the amended and control soils, and therefore only the results of the latter sample have been reported. For both control and MSWC 4.5%, the most abundant minerals detected were silicates and aluminosilicates; indeed, most diffraction peaks testify the prevalent presence of quartz and illite/muscovite, the latter showing less intense peaks in the amended soil (Fig. 4). Moreover, among the phases, the presence of sphalerite (ZnS) is clearly highlighted by some characteristic peaks (e.g. those visible at 2*θ* = 28.56 and 2*θ* = 47.52, 100% and 50% diffraction intensities, respectively). Despite the great similarity between the diffractograms of control and MSWC 4.5% (Fig. 4), some small differences were observed that were associated to changes in the soil mineralogy that occurred over time after the addition of MSWC. First, mineral gypsum (2*θ* = 11.62 and 2*θ* = 20.74) was detected only in the control soil; further, a clear calcite peak at 2*θ* = 29.36 (100% diffraction intensity) was visible in the MSWC-amended soil, while it was weakly visible in the control. No peaks attributable to Pb, As, Cd and Sb mineral phases were identified by XRPD, due to either the low crystallinity of their compounds or to their concentration below the limit of detection of the instrument.

**Fig. 5:**
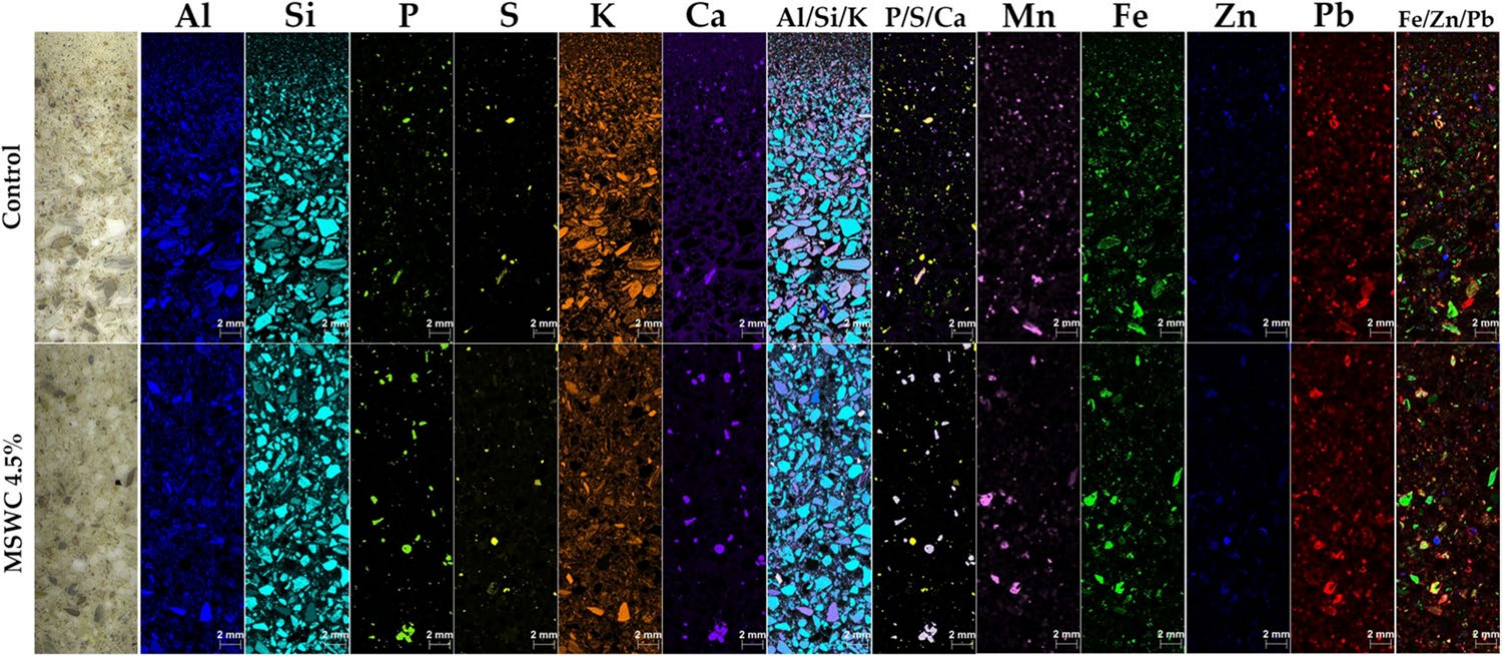
µXRF—Distribution maps of the major elements detected by µXRF in the control and MSWC 4.5% amended soils. Each map has a proper relative intensity colour scale, with brighter colour indicating higher element concentration. In multi-elemental maps, colours should be intended as the combination of the colours of the single-element maps (e.g. in S/ZN/Pb map, violet areas are those in which Zn (blue) and Pb (red) signals overlap)

Therefore, aiming to gain more information on the distribution of PTEs and their fate after treatment with MSWC, a µXRF analysis was carried out (Fig. 5). This technique allows the direct insight into the spatial distribution of elements within the soil solid fraction, thus providing valuable information to assess and/or predict the possible mobilisation of PTEs based on their correlation with other elements (Porfido et al. 2022). Further, as in the case of the present study, µXRF allows visualising changes in the distribution of elements in soil as a consequence of a treatment (i.e. MSWC amendment), with a focus on PTEs. The distribution maps of the main elements detected through µXRF, including Zn and Pb (As, Sb and Cd were below the limit of detection of the instrument) analysis, are shown for control and MSWC 4.5% (Fig. 5). Aluminium, Si, K and Fe were the most abundant elements detected and widely distributed over the soil sections, being the main constituents of the mineral fraction. On the basis of the XRPD results, the portions of the slabs where the solely Si is detected were ascribed to quartz grains. Instead, when Si correlates with Al and K (see Al/Si/K multilayer distribution map in Fig. 5), its presence was attributed to aluminosilicates, e.g. illite-muscovite and feldspars.

In both soils, sulphur showed noticeable correlations with Zn and, although to a lesser extent, with Pb (Fig. S3). The S/Ca correlation was yet found only in the control soil (Fig. S3): indeed, there is no longer coincidence between S and Ca signals in MSWC 4.5% µXRF maps (Fig. 5). As such, µXRF confirms the presence of gypsum only in the control soil (as expected from XRPD results) and the loss of such phase after the MSWC addition. Gypsum has been described in a previous study as a secondary mineral at the Argentiera site (Ara et al. 2013). In both soils, Ca correlated also with P (Fig. 5 and Fig. S3): this indicates that also Ca-phosphates were present, even if not previously detected by XRPD. In the amended soil, a higher concentration of Ca-P-rich particles can be seen (Fig. 5) that along with other smaller particles in which Ca did not correlate with any other detected element (thus explainable as Ca-carbonate or as Ca-rich organic matter) were probably introduced with the compost. Mn, Fe, Zn and Pb were concentrated in grains that appeared darker at the light microscope and, in many cases, formed encrustations around quartz or silicate grains. Sulphur correlation with Zn and Pb is attributable to sulphides, such as sphalerite (as observed by the XRPD technique) and galena, which are typical minerals of the Argentiera mining site, or sulphate as well. Such correlations persist after the amendment (Fig. S3), as expected from XRPD results, which showed the presence of sphalerite also in the MSWC 4.5% soil. In both control and MSWC 4.5% soils, Zn and Pb signals overlap in several soil particles with those of Mn and Fe (Fig. 5), suggesting possible adsorption process on Fe/Mn (hydro)oxides. This latter correlation was likely enhanced in MSWC 4.5%, based on the slightly increase of Zn/Fe and Pb/Mn ratios (Fig. S3). Besides, as it can be observed through the element distribution maps (Fig. 5), in most particles containing Zn and Pb of both treated and untreated soils, these elements do not show correlation with other elements. In such particles, Zn and Pb could therefore occur as carbonates, which are typical weathering phases in sub-alkaline and alkaline soil conditions (C and O are not detectable by µXRF), as well as forming complexes with the oxygenated organic functional groups of MSWC.

Overall, the combination of XRPD and µXRF analysis showed that no changes occurred in PTE spatial distribution and mineralogy within the soil after the MSWC treatment, except for the increase in either the adsorption on Fe–Mn (hydro)oxides and in the complexing with the organic matter introduced. These results are consistent with those get through the sequential extraction analysis (which showed an increase of Zn mobility and suggested the formation of strong complexes between Pb and the organic matter of the MSWC, see paragraph 3.3). Further, an interesting outcome of XRD and µXRF analyses is the loss of Ca-sulphate after the MSWC application to soil. The Ca-sulphate could have been used by the colonising vegetation as a source of Ca and/or by sulphate-reducing soil bacteria.

### Oral, lung and dermal bioaccessibility of PTEs

The bioaccessible fractions of PTEs from the four finer particle-size fractions (20–50, 10–20, 2–10 and < 2 µm) of the control and MSWC-amended soils are reported in Table 2 for Zn, Pb and Cd and Table 3 for Sb and As. The relative bioaccessibility (RB) of these PTEs is provided in Tables S3 and S4. In both the soil samples, the bioaccessibility was higher in the acid fluids (G, ALF and NIHS) compared to neutral mimicking solutions (GI, GAM and CEN) for all the PTEs considered (Tables 2 and 3). Therefore, the pH and the chemical composition (nature and concentration of salts, organic compounds, amino acids, large molecular mass proteins, antioxidants, etc.) of these formulations have strongly affected the bioaccessibility of PTEs (Khelifi et al. 2021). In fact, many organic chemicals of the synthetic fluids can exert a strong complexing capacity towards soil PTEs (Hedberg et al. 2011). A higher bioaccessibility of the PTEs in the acid vs. neutral synthetic formulations was also found by Chaparro Leal et al. (2018) (dermal fluids), Gosselin and Zagury (2020) (respiratory fluids) and Khelifi et al. (2020) (digestive juices). All synthetic formulations extracted greater bioaccessible fractions of each PTE from the fine particle-size fractions (< 2 and 2–10 µm) than from the coarse particles (10–20 and 20–50 µm) (*p* < 0.05, in the most of the cases; Tables 2 and 3). This was basically due to the higher PTE content loaded in the smaller particles, which have a high specific surface area and thus a high capacity to adsorb PTEs (see also column PS-TOT in Tables 2 and 3), as well as a higher concentration of organic matter, Fe/Al oxides and clay minerals and a lower biodurability in the body fluids (Li et al. 2020). These findings make the finer particle-size fractions (< 2 and 2–10 µm) even more dangerous for human health, since they can easily enter the human body through inhalation, dermal contact and accidental ingestion (Kastury et al. 2017).

The amendment of soil with increasing rates of MSWC did not produce significant variations in the bioaccessibility of the PTEs in the soil particle-size fractions, except in a few cases (i.e. Zn, ALF and GAM; Pb, GAM; Sb, GI and NIHS; Tables 2 and 3). Accordingly, the interaction between the two factors, particle size (PS) and compost rate (CR), was rarely statistically significant (except for Zn, GAM; Pb, GAM; Cd, NIHS; Table 2). Similarly, non-significant variations of the PTE bioaccessibility, after application of compost and microbial biostimulant to a contaminated soil of Southern Italy, were also observed by Visconti et al. (2023). The small effect of MSWC on PTE bioaccessibility may be due to the specific extraction conditions, such as very low pH value and 24-h reaction time. Consequently, in the milder extraction, i.e. Gamble’s sub-neutral solution (GAM), a significant effect of MSWC application on Zn and Pb bioaccessibility was observed (Table 2). In these two cases, the higher bioaccessibility was basically associated with small particles (< 2 µm) and medium MSWC rates (3.0–1.5%), while the lower bioaccessibility was observed with coarser particles (20–50 µm to 10–20 µm) and unamended soil (control).

The analysis of the relative bioaccessibility (RB) of PTEs (Tables S3—S4) in relation to the synthetic fluids used showed that the cationic PTEs were more bioaccessible than the anionic ones: Cd (41 and 96% of mean and max RB, respectively; mean and max calculated considering all the 6 synthetic fluids and all the 16 cases deriving from the PS × CR factorial combination) and Zn (mean RB, 34%; max RB, 97%) were the most bioaccessible PTEs; Pb (mean RB, 24%; max RB, 68%) was highly bioaccessible as well. The anionic elements, such as Sb (mean RB, 9%; max RB, 42%) and As (mean RB, 6%; max RB, 19%), were the least bioaccessible (Tables S3-S4). These findings are consistent with those of Khelifi et al. (2020; 2021) and Schaider et al. (2007), which found that Cd was more bioaccessible than Zn and Pb in size-fractionated waste from a mining area of the USA and soil/sediments/tailings from a phosphate-mining area of Tunisia, respectively. Unlike cationic PTEs, anionic PTEs (i.e. Sb and As) are less soluble at acid pH, because they can be stably adsorbed on positively charged minerals, such as Al and Fe (hydr)oxides (Caporale and Violante 2016), and/or can form stable precipitates with soluble Fe^3+^ and/or Al^3+^. This different chemical behaviour can explain, at least in part, the low bioaccessibility of Sb and As, in particular when extracted with acid synthetic fluids (Tables S3-S4). A pH-dependent bioaccessibility of Cd, Pb, Sb and As was also described by Denys et al. (2011) in a validation study of the UBM (i.e. Unified BARGE Method) through a juvenile swine model, on 16 contaminated smelting/mining soils.

Regarding the synthetic fluids, the highest RB was achieved with ALF solution (on average, 51% for all the PTEs, 68% for the cationic PTEs, 24% for the anionic PTEs, mean values calculated considering all the five PTEs or only the cationic or anionic ones and all the 16 cases deriving from the PS × CR factorial combination), followed by G > NIHS > GI > CEN > GAM. The chemical composition and the pH of the synthetic fluids, matched with the reaction time, the chemical properties of the PTEs and possible interactions between them and the added chemicals, are the key factors behind the results obtained (Gosselin and Zagury 2020; Khelifi et al. 2021).

### Risk assessment

The NCR and CR health risks for adults and children due to ingestion, dermal contact and inhalation of bioaccessible fractions of PTEs extracted from medium-fine particle-size fractions (< 10 µm via inhalation, < 50 µm via ingestion and dermal contact) of MSWC-treated and untreated soil samples are provided in Table 4.

**Table 4:** Non-carcinogenic (NCR) and carcinogenic (CR) health risks for adults and children, due to ingestion, dermal contact and inhalation of bioaccessible fractions of PTEs (i.e. Zn, Pb, Cd, Sb and As) extracted from medium-fine particle-size fractions (< 10 µm via inhalation, < 50 µm via ingestion and dermal contact) of control and MSWC-amended soils

Source of variance	Adults						Children					
*NCR*	*HQ As*	*HQ Sb*	*HQ Cd*	*HQ Pb*	*HQ Zn*	*HI PTEs*	*HQ As*	*HQ Sb*	*HQ Cd*	*HQ Pb*	*HQ Zn*	*HI PTEs*
Ingestion	5.5E − 02^a^	2.3E − 01^a^	4.1E − 02^a^	1.1E + 00^a^	4.5E − 02^a^	1.4E + 00^a^	5.1E − 01^a^	2.2E + 00^a^	3.8E − 01^a^	1.0E + 01^a^	4.2E − 01^a^	1.3E + 01^a^
Dermal contact	2.1E − 05^b^	5.9E − 02^b^	1.2E − 02^b^	6.9E − 03^b^	8.1E − 04^b^	7.9E − 02^b^	1.3E − 04^b^	3.8E − 01^b^	8.1E − 02^b^	4.5E − 02^b^	5.3E − 03^b^	5.2E − 01^b^
Inhalation	3.6E − 04^b^	1.8E − 05^c^	2.1E − 08^c^	3.1E − 07^b^	1.7E − 08^b^	3.7E − 04^b^	6.6E − 05^b^	4.7E − 04^c^	1.1E − 05^c^	3.5E − 04^b^	1.7E − 05^b^	9.2E − 04^b^
Exposure route (ER)	***	***	***	***	***	***	***	***	***	***	***	***
Control	2.0E − 02	8.3E − 02	1.9E − 02	3.1E − 01	1.6E − 02	4.5E − 01	1.9E − 01	7.2E − 01	1.7E − 01	2.9E + 00	1.5E − 01	4.1E + 00
MSWC 1.5%	1.9E − 02	1.3E − 01	1.8E − 02	4.3E − 01	1.7E − 02	6.1E − 01	1.8E − 01	1.1E + 00	1.6E − 01	4.0E + 00	1.6E − 01	5.6E + 00
MSWC 3.0%	1.8E − 02	1.1E − 01	1.6E − 02	4.0E − 01	1.3E − 02	5.5E − 01	1.7E − 01	9.4E − 01	1.4E − 01	3.7E + 00	1.2E − 01	5.1E + 00
MSWC 4.5%	1.5E − 02	8.7E − 02	1.7E − 02	3.4E − 01	1.5E − 02	4.7E − 01	1.3E − 01	7.7E − 01	1.5E − 01	3.2E + 00	1.4E − 01	4.3E + 00
MSWC rate (CR)	ns	ns	ns	ns	ns	ns	ns	ns	ns	ns	ns	ns
ER × CR	ns	ns	ns	ns	ns	ns	ns	ns	ns	ns	ns	ns
*CR*	*CR As*	*CR Sb*	*CR Cd*	*CR Pb*	*CR Zn*	*CR PTEs*	*CR As*	*CR Sb*	*CR Cd*	*CR Pb*	*CR Zn*	*CR PTEs*
Ingestion	1.1E − 05^a^	-	1.1E − 04	4.4E − 01	-	4.4E − 01^a^	1.0E − 04^a^	-	1.0E − 03	4.1E + 00	-	4.1E + 00^a^
Dermal contact	6.9E − 10^b^	-	-	-	-	6.9E − 10^b^	4.5E − 09^b^	-	-	-	-	4.5E − 09^b^
Inhalation	1.9E − 10^b^	-	6.1E − 10	1.1E − 05	-	1.1E − 05^b^	5.3E − 10^b^	-	1.7E − 09	2.9E − 05	-	2.9E − 05^b^
Exposure route (ER)	***		***	***		***	***		***	***		***
Control	4.0E − 06	-	5.8E − 05	1.9E − 01	-	1.3E − 01	3.7E − 05	-	5.4E − 04	1.8E + 00	-	1.2E + 00
MSWC 1.5%	3.8E − 06	-	5.5E − 05	2.6E − 01	-	1.8E − 01	3.6E − 05	-	5.1E − 04	2.5E + 00	-	1.6E + 00
MSWC 3.0%	3.5E − 06	-	4.7E − 05	2.5E − 01	-	1.6E − 01	3.3E − 05	-	4.4E − 04	2.3E + 00	-	1.5E + 00
MSWC 4.5%	2.9E − 06	-	5.2E − 05	2.1E − 01	-	1.4E − 01	2.7E − 05	-	4.9E − 04	1.9E + 00	-	1.3E + 00
MSWC rate (CR)	*ns*		*ns*	*ns*		*ns*	*ns*		*ns*	*ns*		*ns*
ER × CR	*ns*		*ns*	*Ns*		*ns*	*ns*		*ns*	*ns*		*ns*

Exposure route (ER), compost rate (CR) and their interactions were compared by two-way ANOVA, Duncan’s multiple range test (*p < 0.05; **p < 0.01; ***p < 0.001; *ns* not significant). Different lowercase letters within each column indicate significant differences (*p* < 0.05). *HQ* hazard quotient, *HI* Hazard Index

Data analysis highlighted that children exposed to soil particulate matter from Argentiera mining site are subject to higher NCR and CR risks than adults (Table 4). This is basically due to the relatively higher dose of PTEs that can enter a lighter human body, the higher breathing frequency, the duration of outdoor activities and so on (Buonanno et al. 2013; Yang et al. 2014). According to the U.S. EPA–based risk assessment, oral ingestion was the riskiest exposure way, as it produces a significantly higher hazard quotient (HQ) (Supplementary text) and CR than dermal contact and inhalation (Table 4). Similarly, the ingestion of PTEs was the route that primarily contributed to rise of NCR and CR risks in similar case studies (Khelifi et al. 2021; Visconti et al. 2023; Yu and Yang 2019; Zhao et al. 2014).

Among the PTEs, Pb showed the highest HQ (for both adults and children, considering all the exposure routes and all the experimental treatments; Table 4), because of its high toxicity (leading to low reference values) and high content in fine particle-size fractions; then, Sb was the second most risky PTE, followed by Cd and As which contributed similarly to the overall NCR and CR risks (Table 4). The ingestion HQ of Zn (the most abundant element in the site) was also consistent with that of Cd and As, while the dermal and inhalation HQs were lower in comparison to the other more toxic PTEs.

The sum of the ingestion HQs of each PTE resulted in a Hazard Index (HI) greater than 1, namely a trigger value indicating possible serious non-carcinogenic effects on human health (US EPA 2011). Accordingly, the sum of the ingestion CR of each PTE also resulted in values of concern (magnitude of 10–1). In general, CR values lower than 10–6 are considered negligible, while those above 10–4 are considered harmful and can lead to serious carcinogenic effects on human health (US EPA 2011). The worrying results of the risk assessment were largely affected by the parameters associated with each exposure pathway (US EPA 2011) and the PTE-specific reference values (related to the toxicity of each PTE). In other words, the real risks to human health are probably lower than those estimated by the risk assessment (Table 4), if we consider the nature, geochemical distribution and mobility of the PTEs analysed in the study area (Figs. 2 and 3).

## Conclusions

The results obtained from this study showed that the addition of MSWC to a soil contaminated by PTEs can represent an effective and lasting environmental management strategy. The addition of MSWC had a positive impact on soil fertility (e.g. increased organic matter, extractable P and CEC); reduced the pool of more labile As, Sb and Cd and increased the residual fraction of Sb, As and Zn, suggesting the formation of stable and long-lasting interactions between MSWC and PTEs. The information obtained from sequential extractions (i.e. PTE mobility) agreed with that resulting from mineralogical analyses, where it is interesting to note, after 6 years of compost addition, the loss of the gypsum phase in amended soils. The bioaccessibility of PTEs was greater in acidic fluids (i.e. G, ALF and NIHS) than in neutral mimicking solutions (i.e. GI, GAM and CEN) in both treated and untreated soils, highlighting how the pH and chemical composition of the formulations have mainly affected the bioaccessibility. According to risk assessment data, children exposed to soil particulate matter from the Argentiera mine site are subject to higher NCR and CR risks than adults. Moreover, HI greater than 1 (due to PTE ingestion) highlighted possible serious non-cancer effects on human health, while also cancer risks were of concern. The addition of the amendment did not significantly reduce the risk, as the relative bioaccessibility of the contaminants did not decrease in the amended soils.

Overall, the results presented indicate that the addition of MSWC can be effectively used to limit the long-term spread of PTEs from a contaminated soil. This would promote plant growth and the restoration of soil functionality, which are essential for a complete remediation of the contaminated site. The addition of MSWC at 4.5% proved to be the most effective treatment in decreasing the mobility of PTEs, particularly As and Sb. Furthermore, MSWC was effective at reducing PTE mobility over time with a single application, proving that the treatment employed is cost-effective. Indeed, the extent of the contaminated area often does not make compost addition sustainable, especially if repeated applications over time are necessary. Although the addition of MSWC is a key intervention to reduce soil erosion and PTE dispersion, it showed a limited impact in the long term on mitigating the contaminants bioaccessibility and the related health risks. For this reason, the provision of monitoring plan for the risk assessment is imperative.

### Supplementary Information

Below is the link to the electronic supplementary material.

Supplementary file1 (PPTX 1565 KB)

Supplementary file2 (DOCX 51 KB)

Supplementary file3 (DOCX 21 KB)

## Data Availability

Data will be made available on request.
