# Supplementary material for: Long-term effect of municipal solid waste compost on the recovery of a potentially toxic element (PTE)-contaminated soil: PTE mobility, distribution and bioaccessibility
Source: Environ Sci Pollut Res Int. 2023 Nov 18;30(58):122858–74. doi: 10.1007/s11356-023-30831-y (PMC10724333; doi:10.1007/s11356-023-30831-y)
Supplement: Supplementary file 1 — Supplementary file1 (PPTX 1565 KB) [file 11356_2023_30831_MOESM1_ESM.pptx]

## Slide 1
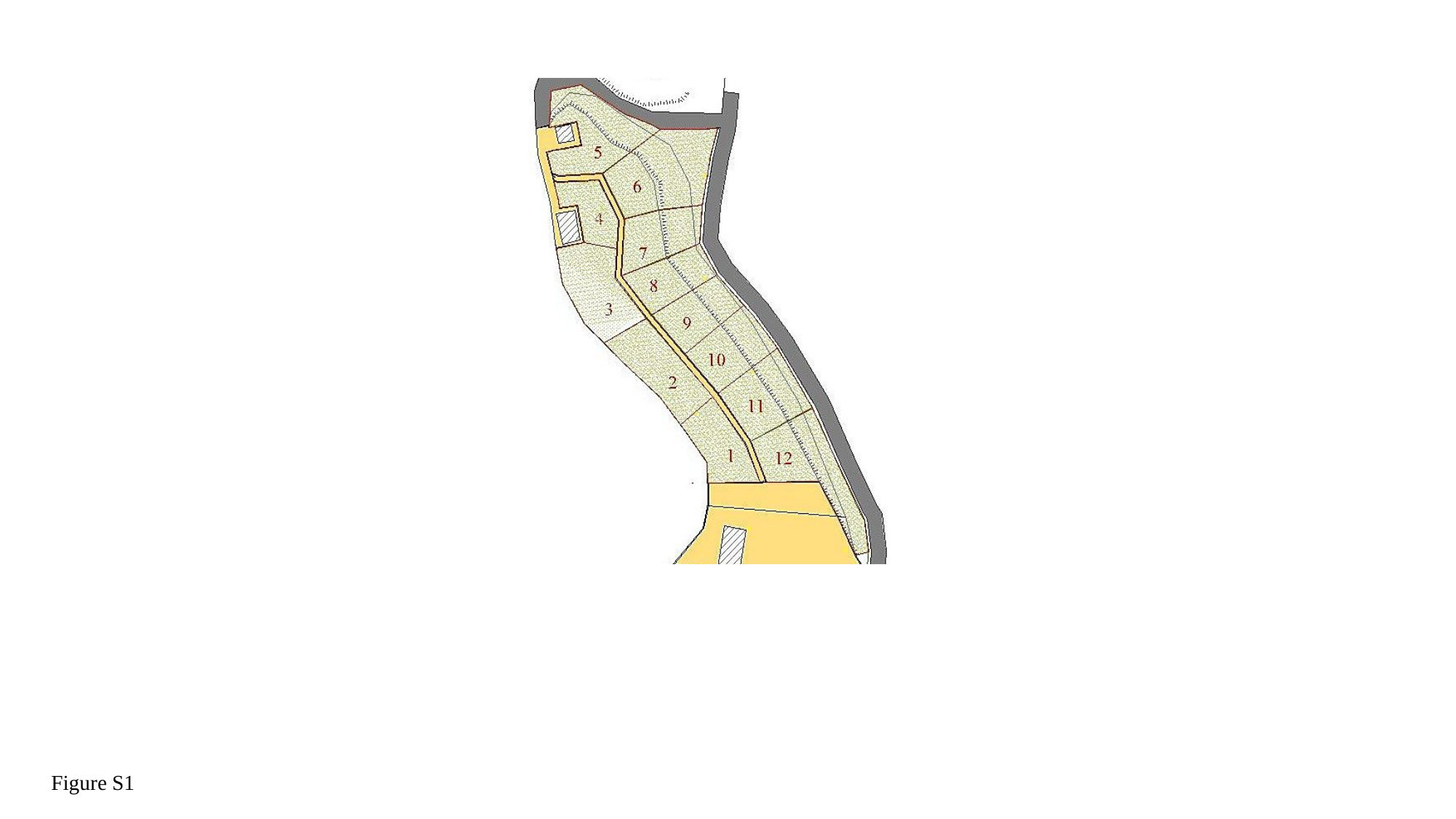

Figure S1

## Slide 2
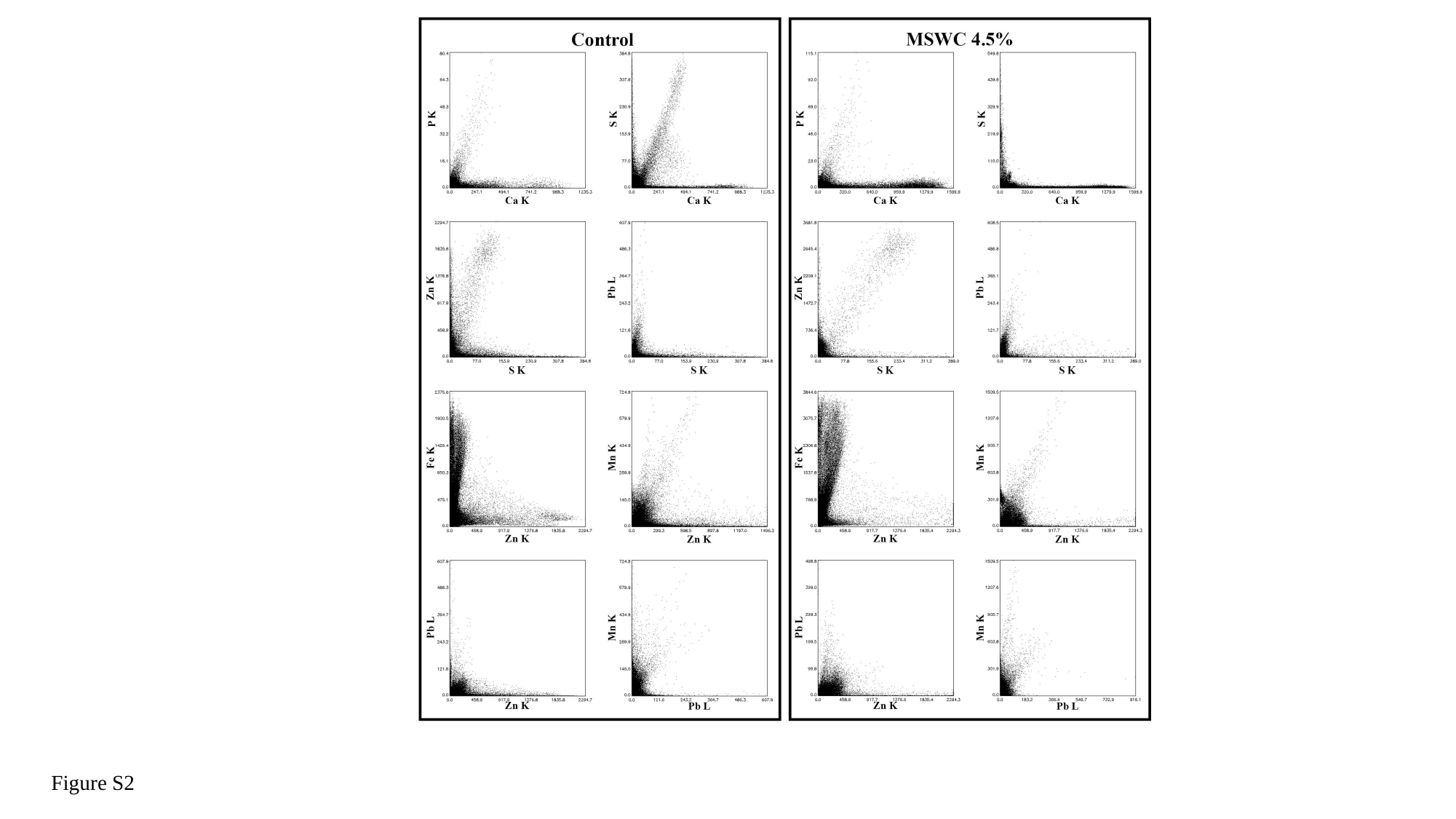

Figure S2

## Slide 3
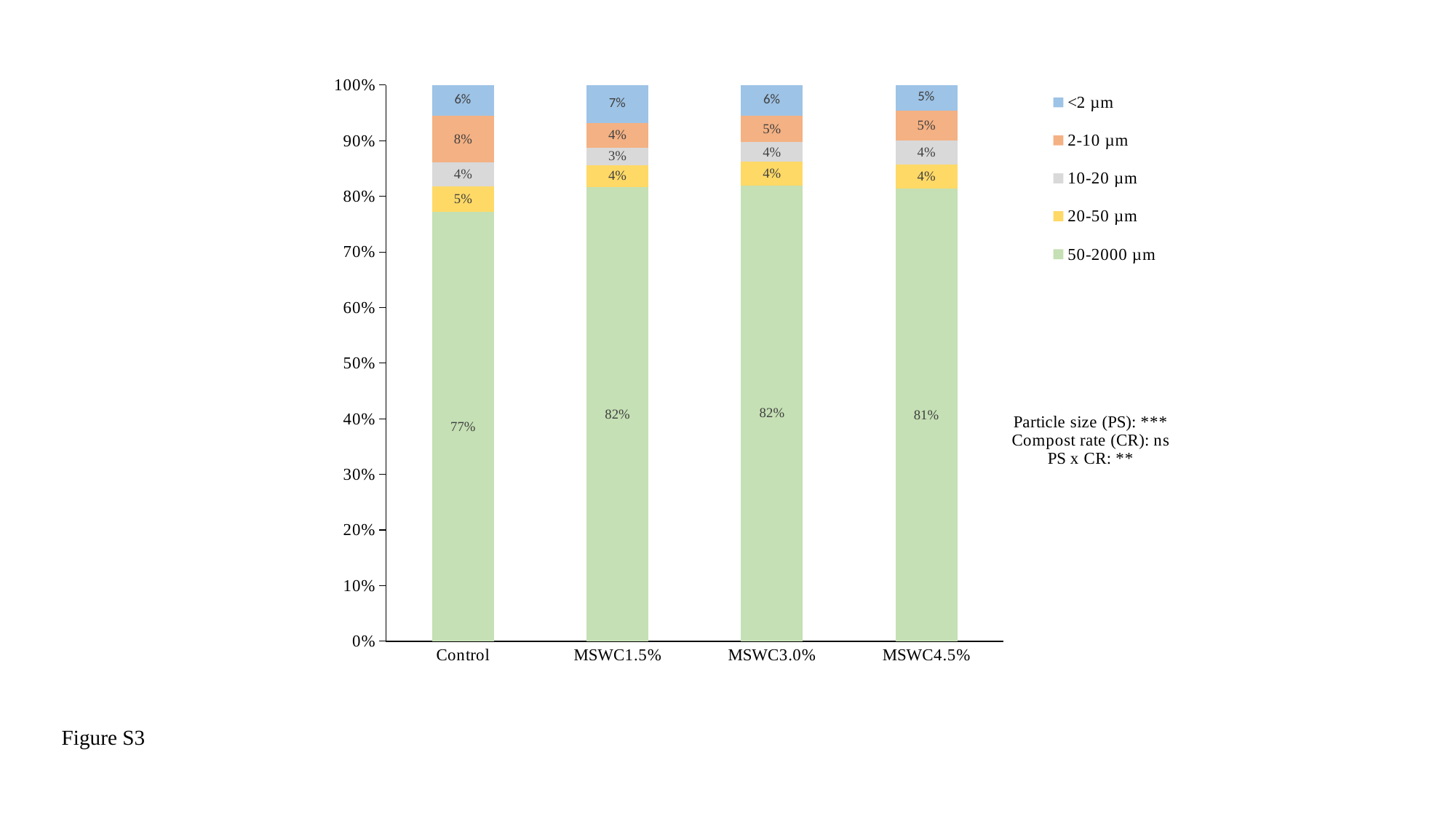

### Chart: Particle size (PS): ***
Compost rate (CR): ns
PS x CR: **
| Category | 50-2000 µm | 20-50 µm | 10-20 µm | 2-10 µm | <2 µm |
|---|---|---|---|---|---|
| Control | 0.7715121256174831 | 0.046088544766583826 | 0.043843305105201735 | 0.0828063122152558 | 0.05574971229547551 |
| MSWC1.5% | 0.8169594463677201 | 0.03946263585629024 | 0.031301384092626644 | 0.0443414096470999 | 0.06793512403626298 |
| MSWC3.0% | 0.8195638158222178 | 0.04267734147919573 | 0.03523730340434007 | 0.046936624278319766 | 0.05558491501592678 |
| MSWC4.5% | 0.814304970055691 | 0.04340894788870843 | 0.04255221237146077 | 0.05418551077022527 | 0.045548358913914594 |Figure S3
