# Supplementary material for: Long-term effect of municipal solid waste compost on the recovery of a potentially toxic element (PTE)-contaminated soil: PTE mobility, distribution and bioaccessibility
Source: Environ Sci Pollut Res Int. 2023 Nov 18;30(58):122858–74. doi: 10.1007/s11356-023-30831-y (PMC10724333; doi:10.1007/s11356-023-30831-y)
Supplement: Supplementary file 2 — Supplementary file2 (DOCX 51 KB) [file 11356_2023_30831_MOESM2_ESM.docx]

**Table S1** Characteristics of MSWC used (dry matter basis)

| Chemical parameters | MSWC |
| --- | --- |
| pH_(H2O)_ | 8.60 ± 0.02 |
| EC (mS cm^-1^) | 4.59 ± 0.01 |
| pH_PZC_ | 4.47±0.06 |
| Organic matter (%) | 45.22 ±4.06 |
| Dissolved organic carbon (DOC, mg kg^-1^) | 0.603 ±0.04 |
| Cation exchange capacity (cmol_(+)_ kg^-1^) | 77.75±0.21 |
| Elemental composition C (%) | 26.38±0.39 |
| Elemental composition N (%) | 2.80±0.07 |
| Elemental composition H (%) | 3.20±0.01 |
| Total extractable carbon (TEC, %) | 13.34±0.58 |
| Humic acids (HA, %) | 11.18±0.25 |
| HA Total Acidity (cmol_(+)_ kg^-1^) | 9.43±0.44 |
| HA COOH (cmol_(+)_ kg^-1^) | 5.86±0.53 |
| HA Ar-OH (cmol_(+)_ kg^-1^) | 3.57±0.39 |
| Fulvic acids (FA, %) | 1.01±0.13 |
| FA Total Acidity (cmol_(+)_ kg^-1^) | 13.41±1.53 |
| FA COOH (cmol_(+)_ kg^-1^) | 11.61±1.42 |
| FA Ar-OH (cmol_(+)_ kg^-1^) | 1.81±0.07 |
| *Total elements (mg kg^-1^)* |  |
| Sb | n.d. |
| Zn | 26.91±0.89 |
| Pb | 3.72±0.15 |
| Cd | n.d. |
| Cu | n.d. |
| Fe | 5494±66.0 |
| Mn | 147.9±6.2 |
| Na | 2534±28.5 |
| K | 2780±24.0 |
| Mg | 4504±121 |
| Ca | 80,113±122 |

*n.d.=not determined.*

**Table S2** Pseudo-total (PS-TOT) concentrations of Zn, Pb, Cd, Sb and As in the 50-2000 µm particle-size fraction of control and MSWC amended soils

|  |  | Zn | Pb | Cd | Sb | As |
| --- | --- | --- | --- | --- | --- | --- |
| Particle size | Compost rate | mg kg^-1^ | | | | |
| 50-2000 µm | Control | 8560^c^ | 2600^a^ | 24.2^c^ | 237^a^ | 30.1^b^ |
|  | MSWC 1.5 % | 8381^c^ | 2551^a^ | 21.5^d^ | 198^c^ | 16.8^d^ |
|  | MSWC 3.0 % | 10067^a^ | 1768^b^ | 30.0^a^ | 225^b^ | 25.1^c^ |
|  | MSWC 4.5 % | 9214^b^ | 1745^b^ | 27.9^b^ | 219^b^ | 32.2^a^ |
|  | *Significance* | ***** | ***** | ***** | ***** | ***** |

*For the sake of clarity, this wide table shows only the mean values, not followed by standard errors. Compost rate (CR) was compared by one-way ANOVA, Duncan’s multiple-range test (* p<0.05; ** p<0.01; *** p<0.001; ns: not significant). Different lowercase letters within each column indicate significant differences (p<0.05).*

**Table S3** Relative bioaccessibility of Zn, Pb and Cd in particle-size fractions of control and MSWC amended soils: gastric (G) and gastro-intestinal (GI) solutions, lysosomal (ALF), and lung interstitial (GAM) fluids, acid (NIHS) and neutral (CEN) synthetic sweats

|  | G | GI | ALF | GAM | NIHS | CEN | |
| --- | --- | --- | --- | --- | --- | --- | --- |
|  | % of the total content | | | | | |  |
| **Zn** |  |  |  |  |  |  | |
| <2 µm | 52^ab^ | 26 | 78^a^ | 0.17^a^ | 69^ab^ | 0.5 | |
| 2-10 µm | 56^a^ | 27 | 79^a^ | 0.11^b^ | 79^a^ | 1.6 | |
| 10-20 µm | 47^bc^ | 22 | 67^b^ | 0.10^b^ | 60^b^ | 0.4 | |
| 20-50 µm | 40^c^ | 17 | 57^c^ | 0.09^b^ | 45^c^ | 0.2 | |
| *Particle size (PS)* | *** | *ns* | **** | *** | *** | *ns* | |
|  |  |  |  |  |  |  | |
| Control | 51 | 22 | 73 | 0.07^c^ | 67 | 0.5 | |
| MSWC 1.5 % | 47 | 24 | 68 | 0.13^b^ | 64 | 1.1 | |
| MSWC 3.0 % | 48 | 25 | 66 | 0.24^a^ | 62 | 1.0 | |
| MSWC 4.5 % | 48 | 23 | 72 | 0.09^bc^ | 55 | 0.3 | |
| *Compost rate (CR)* | *ns* | *ns* | *ns* | **** | *ns* | *ns* | |
|  |  |  |  |  |  |  | |
| *PS x CR* | *ns* | *ns* | *ns* | *** | *ns* | *ns* | |
|  |  |  |  |  |  |  | |
| **Pb** |  |  |  |  |  |  | |
| <2 µm | 47^b^ | 9 | 60 | 0.13^a^ | 13 | 0.04 | |
| 2-10 µm | 55^a^ | 14 | 65 | 0.06^b^ | 19 | 0.05 | |
| 10-20 µm | 55^a^ | 18 | 65 | 0.09^b^ | 20 | 0.04 | |
| 20-50 µm | 53^a^ | 17 | 62 | 0.07^b^ | 15 | 0.02 | |
| *Particle size (PS)* | *** | *ns* | *ns* | **** | *ns* | *ns* | |
|  |  |  |  |  |  |  | |
| Control | 52 | 11 | 63 | 0.05^b^ | 17 | 0.04 | |
| MSWC 1.5 % | 52 | 17 | 62 | 0.08^b^ | 17 | 0.07 | |
| MSWC 3.0 % | 56 | 19 | 62 | 0.18^a^ | 18 | 0.04 | |
| MSWC 4.5 % | 51 | 15 | 65 | 0.06^b^ | 15 | 0.02 | |
| *Compost rate (CR)* | *ns* | *ns* | *ns* | **** | *ns* | *ns* | |
|  |  |  |  |  |  |  | |
| *PS x CR* | *ns* | *ns* | *ns* | *** | *ns* | *ns* | |
|  |  |  |  |  |  |  | |
| **Cd** |  |  |  |  |  |  | |
| <2 µm | 66^a^ | 48^a^ | 81^a^ | 0.7 | 81^a^ | 3.6 | |
| 2-10 µm | 69^a^ | 48^a^ | 80^a^ | 0.7 | 84^a^ | 8.4 | |
| 10-20 µm | 54^b^ | 39^b^ | 67^b^ | 0.6 | 67^b^ | 2.9 | |
| 20-50 µm | 46^b^ | 32^c^ | 58^b^ | 0.5 | 52^c^ | 1.5 | |
| *Particle size (PS)* | **** | **** | *** | *ns* | ***** | *ns* | |
|  |  |  |  |  |  |  | |
| Control | 62 | 44 | 75 | 0.5^b^ | 75^a^ | 3.6 | |
| MSWC 1.5 % | 54 | 39 | 68 | 0.7^ab^ | 69^ab^ | 6.0 | |
| MSWC 3.0 % | 59 | 43 | 68 | 0.9^a^ | 73^a^ | 5.1 | |
| MSWC 4.5 % | 58 | 39 | 72 | 0.5^b^ | 63^b^ | 2.1 | |
| *Compost rate (CR)* | *ns* | *ns* | *ns* | *** | *** | *ns* | |
|  |  |  |  |  |  |  | |
| *PS x CR* | *ns* | *ns* | *ns* | *ns* | *** | *ns* | |

*For the sake of clarity, this wide table shows only the mean values, not followed by standard errors. Particle size (PS), Compost rate (CR) and their interactions were compared by two-way ANOVA, Duncan’s multiple-range test (* p<0.05; ** p<0.01; *** p<0.001; ns: not significant). Different lowercase letters within each column indicate significant differences (p<0.05).*

**Table S4** Relative bioaccessibility of Sb and As in particle-size fractions of control and MSWC amended soils: gastric (G) and gastro-intestinal (GI) solutions, lysosomal (ALF), and lung interstitial (GAM) fluids, acid (NIHS) and neutral (CEN) synthetic sweats

|  | G | GI | ALF | GAM | NIHS | CEN | |
| --- | --- | --- | --- | --- | --- | --- | --- |
|  | % of the total content | | | | | |  |
| **Sb** |  |  |  |  |  |  | |
| <2 µm | 1.8 | 4.0^c^ | 37^a^ | 0.40 | 7^b^ | 0.08 | |
| 2-10 µm | 2.1 | 6.1^b^ | 36^a^ | 0.30 | 12^a^ | 0.09 | |
| 10-20 µm | 2.4 | 8.1^a^ | 33^ab^ | 0.36 | 15^a^ | 0.11 | |
| 20-50 µm | 2.2 | 8.2^a^ | 28^b^ | 0.33 | 11^a^ | 0.18 | |
| *Particle size (PS)* | *ns* | **** | *** | *ns* | *** | *ns* | |
|  |  |  |  |  |  |  | |
| Control | 2.0 | 6.0 | 34 | 0.37 | 11 | 0.13 | |
| MSWC 1.5 % | 2.3 | 7.4 | 35 | 0.27 | 13 | 0.08 | |
| MSWC 3.0 % | 2.4 | 7.2 | 32 | 0.33 | 11 | 0.09 | |
| MSWC 4.5 % | 1.9 | 6.3 | 33 | 0.39 | 10 | 0.14 | |
| *Compost rate (CR)* | *ns* | *ns* | *ns* | *ns* | *ns* | *ns* | |
|  |  |  |  |  |  |  | |
| *PS x CR* | *ns* | *ns* | *ns* | *ns* | *ns* | *ns* | |
|  |  |  |  |  |  |  | |
| **As** |  |  |  |  |  |  | |
| <2 µm | 6.0^c^ | 3.3^c^ | 16^a^ | 2.5 | 0.32 | 0.13 | |
| 2-10 µm | 8.7^a^ | 9.5^b^ | 17^a^ | 2.2 | 0.25 | 0.19 | |
| 10-20 µm | 9.3^a^ | 13^a^ | 16^a^ | 1.9 | 0.55 | 0.32 | |
| 20-50 µm | 7.5^b^ | 9.8^b^ | 12^b^ | 1.6 | 0.34 | 0.32 | |
| *Particle size (PS)* | **** | ***** | **** | *ns* | *ns* | *ns* | |
|  |  |  |  |  |  |  | |
| Control | 7.7 | 8.3 b | 16 | 2.5 | 0.38 | 0.17 | |
| MSWC 1.5 % | 8.5 | 9.9 a | 16 | 1.6 | 0.39 | 0.23 | |
| MSWC 3.0 % | 8.0 | 9.3 a | 15 | 1.7 | 0.57 | 0.30 | |
| MSWC 4.5 % | 7.5 | 8.0 b | 14 | 2.1 | 0.11 | 0.33 | |
| *Compost rate (CR)* | *ns* | **** | *ns* | *ns* | *ns* | *ns* | |
|  |  |  |  |  |  |  | |
| *PS x CR* | *ns* | **** | *ns* | *ns* | *ns* | *ns* | |

*For the sake of clarity, this wide table shows only the mean values, not followed by standard errors. Particle size (PS), Compost rate (CR) and their interactions were compared by two-way ANOVA, Duncan’s multiple-range test (* p<0.05; ** p<0.01; *** p<0.001; ns: not significant). Different lowercase letters within each column indicate significant differences (p<0.05).*

**Table S5** Parameters and reference doses of the health risk assessment (Reference: US.EPA, 2011; Khelifi et al., 2021)

| Parameter | | | Unit | | | Adult | | | | Children | | |
| --- | --- | --- | --- | --- | --- | --- | --- | --- | --- | --- | --- | --- |
| IR: Inhalation rate | | | | m^3^day^-1^ | | 12.8 | | | | | 7.63 | |
| IR: ingestion rate | | | | mg day^-1^ | | 100 | | | | | 200 | |
| EF: exposure frequency | | | | day·year^-1^ | | 350 | | | | | 350 | |
| ED: exposure duration | | | | years | | 24 | | | | | 6 | |
| BW: body weight | | | | kg | | 70 | | | | | 15 | |
| AT: averaged time (ED) | | | | days | | 8760 | | | | | 2190 | |
| PEF: Particle Emission Factor | | | | m^3^ kg^-1^ | | 1.36E+09 | | | | | 1.36E+09 | |
| SL: skin adherence factor | | | | mg·cm^-2^·day^-1^ | | 0.07 | | | | | 0.2 | |
| SA: skin area | | | | cm^2^ day^-1^ | | | 5700 | | | | | 2800 |
| ABS: dermal absorption factor | | | | unitless | | | 0.001 | | | | | 0.001 |
|  | | | | |  | | |  |  | | | |
|  | Zn | Pb | | | Cd | | | Sb | | | As | |
|  | mg kg^-1^ day^-1^ | | | | | | | | | | | |
| RfD _ing_ | 3.00E-01 | 3.52E-03 | | | 1.00E-03 | | | 4.00E-04 | | | 3.00E-04 | |
| CSF _ing_ | - | 8.50E-03 | | | 3.80E-01 | | | - | | | 1.50E+00 | |
| RfD _derm_ | 6.00E-02 | 5.25E-04 | | | 1.00E-05 | | | 8.00E-06 | | | 1.23E-04 | |
| CSF _derm_ | - | - | | | - | | | - | | | 3.66E+00 | |
| RfD _inh_ | 3.00E-01 | 3.50E-03 | | | 1.00E-03 | | | 4.00E-04 | | | 1.23E-04 | |
| CSF _inh_ | - | 4.20E-02 | | | 6.30E+00 | | | - | | | 1.51E+01 | |
