# Supplementary material for: Long-term effect of municipal solid waste compost on the recovery of a potentially toxic element (PTE)-contaminated soil: PTE mobility, distribution and bioaccessibility
Source: Environ Sci Pollut Res Int. 2023 Nov 18;30(58):122858–74. doi: 10.1007/s11356-023-30831-y (PMC10724333; doi:10.1007/s11356-023-30831-y)
Supplement: Supplementary file 3 — Supplementary file3 (DOCX 21 KB) [file 11356_2023_30831_MOESM3_ESM.docx]

*Equations applied in the health risk assessment (Reference: US.EPA, 2011; Khelifi et al., 2021)*

The average daily dose (ADD) of a PTE received by a person through ingestion, inhalation and dermal contact of airborne soil particulate was calculated using the equations 1, 2 and 3, respectively.

$${ADD}_{\mathrm{ingestion}}=C\times\frac{IR\times EF\times ED}{BW\times AT}\times{10}^{-6} (Eq1)$$

$${ADD}_{\mathrm{inhalation}}=C\times\frac{IR\times EF\times ED}{PEF\times BW\times AT} (Eq2)$$

$${ADD}_{\mathrm{dermal}}=C\times\frac{SL\times SA\times ABS\times EF\times ED}{BW\times AT}\times{10}^{-6} (Eq3)$$

where, C is the PTE bioaccessible fraction, IR is the ingestion or inhalation rate, EF is the exposure frequency, ED is the exposure duration, BW is the body weight, AT is the averaged time (365 x ED), PEF is the particle emission factor, SL is the skin adherence factor, SA is the exposed skin surface area, and ABS is the dermal absorption factor.

Subsequently, the ADD for each exposure pathway was divided by the corresponding reference dose (RfD) to yield a Hazard Quotient (HQ; equations 4, 5 and 6) for systemic toxicity.

$${HQ}_{\mathrm{ing}}=\frac{\mathrm{ADD}_{ing}}{\mathrm{RfD}_{ing}} (Eq4)$$

$${HQ}_{\mathrm{inh}}=\frac{\mathrm{ADD}_{inh}}{\mathrm{RfD}_{inh}} (Eq5)$$

$${HQ}_{\mathrm{der}}=\frac{\mathrm{ADD}_{der}}{\mathrm{RfD}_{der}} (Eq6)$$

Assuming that the non-cancerogenic risks of the single PTEs are additive, we calculated the cumulative non-carcinogenic risk (NCR) expressed as the Hazard Index (HI; equation 7):

$$NCR=HI=\sum HQs (Eq 7)$$

To assess the carcinogenic risks (CR), the different ADD were multiplied by the corresponding Cancer Slope Factor (CSF) to produce a level of excess lifetime cancer risk (equation 8) induced by ingestion, inhalation and dermal contact of contaminated soil particulate.

$$CR=ADD\times CSF (Eq 8)$$

Carcinogenic risk expressed as the total cancer risk is the sum of CR of all the exposure routes (equation 9)

$${CR}_{\mathrm{total}}= {CR}_{\mathrm{ing}}+ {CR}_{\mathrm{inh}}+ {CR}_{\mathrm{der}} (Eq 9)$$
